# Mechanical Thrombectomy in Stroke. Experience from Switching from Stent Retriever Only to Stent Retriever Combined with Aspiration Catheter

**DOI:** 10.3390/jcm10091802

**Published:** 2021-04-21

**Authors:** Grzegorz Meder, Paweł Żuchowski, Wojciech Skura, Violetta Palacz-Duda, Milena Świtońska, Magdalena Nowaczewska, Paweł Sokal

**Affiliations:** 1 Department of Interventional Radiology, Jan Biziel University Hospital No. 2, Ujejskiego 75 Street, 85-168 Bydgoszcz, Poland; wojciechskura@wp.pl; 2 Department of Rheumatology and Connective Tissue Diseases, Jan Biziel University Hospital No. 2, Ujejskiego 75 Street, 85-168 Bydgoszcz, Poland; pzuchowski81@gmail.com; 3 Stroke Intervention Centre, Department of Neurosurgery and Neurology, Jan Biziel University Hospital No. 2, Ujejskiego 75 Street, 85-168 Bydgoszcz, Poland; violkapduda1@tlen.pl (V.P.-D.); m.switonska@cm.umk.pl (M.Ś.); 4 Department of Neurosurgery and Neurology, Faculty of Health Sciences, Nicolaus Copernicus University in Toruń, Ludwik Rydygier Collegium Medicum, Ujejskiego 75 Street, 85-168 Bydgoszcz, Poland; pawel.sokal@cm.umk.pl; 5 Department of Otolaryngology, Head and Neck Surgery, and Laryngological Oncology, Ludwik, Rydygier Collegium Medicum in Bydgoszcz, Nicolaus Copernicus University, M. Skłodowskiej—Curie 9, 85-090 Bydgoszcz, Poland; m.nowaczewska@cm.umk.pl

**Keywords:** mechanical thrombectomy, stent retriever, stentriever, aspiration catheter, ischemic stroke, large vessel occlusion, first pass effect

## Abstract

Endovascular treatment is a rapidly evolving technique; therefore, there is a constant need to evaluate this method and its modifications. This paper discusses a single-center experience and the results of switching from the stent retriever only (SO) mechanical thrombectomy (MT) to the combined approach (CA), with a stent retriever and aspiration catheters. Methods: The study involved a retrospective analysis of 70 patients undergoing MT with the use of either SO or CA. The primary endpoint was the frequency of perfect reperfusion defined as grade 3 of the modified Thrombolysis in Cerebral Infarction scale (mTICI) after the first pass. The secondary endpoints were the procedure success, defined as mTICI grades 2b-3; time of the procedure; clinical outcome, measured by 90 days’ modified Rankin Scale (mRS) score; Δ NIHSS, defined as the difference between National Institutes of Health Stroke Scale (NIHSS) score at patients’ admission and discharge; and the total number of device passes. Results: Out of the 70 patients included, 33 were treated with SO and 37 with CA. In both groups, a total number of 42 patients received intravenous recombined tissue plasminogen activator (iv-rTPA: 20 patients (60.6%) in the SO group and 22 patients (59.5%) in the CA group (*p* = 1.000). There was a significant difference between the groups regarding first-pass success rate, with 46% in the CA group and 18% in the SO group, (OR 3.83, 95% CI 1.28 to 11.44, *p* = 0.016). Complete procedure success tended to be more frequent in the CA group than in the SO group—94.6% vs. 84.8% (OR 3.13, 95% CI 0.56 to 17.34, *p* = 0.193)—and CA tended to require a lower number of passes than SO (mean 1.76 vs. 2.09 passes per procedure, *p* = 0.114), yet these differences did not reach statistical significance. Mean duration of the procedure was significantly shorter in the CA group than in the SO group (49 min vs. 64 min, *p* = 0.017). There was a significant difference in clinical outcomes, with higher Δ NIHSS (9.3 in the CA group vs. 6.7 in the SO group, *p* = 0.025) after the procedure and 90-day mRS (median 2 in the CA group vs. 4 in the SO group, *p* = 0.031). Conclusions: Combining stent retrievers with aspiration catheters may offer a beneficial effect on angiographic results and clinical outcomes in stroke patients undergoing endovascular treatment.

## 1. Introduction

In recent years, mechanical thrombectomy (MT) has become the standard of care in stroke caused by large vessel occlusion (LVO), as multiple randomized clinical trials proved its superiority over the best medical treatment [1,2,3]. Endovascular treatment (EVT) is a rapidly evolving technique; therefore, there is a constant need to evaluate this method and its modifications. Blood flow restoration in the affected vessel can be predominantly obtained by either using a stent retriever or aspiration catheters. A recent randomized trial comparing those two techniques showed comparable results regarding reperfusion grades in the modified Thrombolysis in Cerebral Infarction scale (mTICI) and clinical outcomes measured with the modified Rankin Scale (mRS) at 90 days [4]. At the starting point, we introduced MT using stent retrievers (SR) and large-bore guiding catheters with proximal aspiration. In some cases, only aspiration catheters (AC) were used. Initial outcomes were equally good, yet some recent papers demonstrated promising results when combining these two methods [5,6]. This paper describes our experiences and results of switching from simple stent retriever-based mechanical thrombectomy to the combined approach with a stent retriever and aspiration catheters.

## 2. Materials and Methods

### 2.1. Study Population

This retrospective study included patients, from our prospectively acquired database, who underwent EVT for LVO between June 2019 and April 2020. During this period, we performed 128 EVTs in stroke patients. Only patients who underwent MT, either with a stent retriever or a stent retriever combined with an aspiration catheter, were included in the analysis. We excluded patients treated with aspiration catheters only, patients with posterior circulation strokes, tandem occlusions, dissections, thrombosed stents, and cases when the operator switched the technique (total excluded *n* = 58). In total, 70 patients were included. Out of those patients, 33 were treated with stent retriever only (SO) and 37 with the combined approach (CA). A total number of 42 patients received intravenous recombined tissue plasminogen activator (iv-rTPA) in both groups. There was no difference in the administration of iv-rTPA between both groups (20 patients, 60.6% in the SO group vs. 22 patients, 59.5% in the CA group; *p* = 1.000).

Consent for treatment was obtained from the patients, in compliance with national guidelines. The study was approved by the local Institutional Review Board. The study was conducted in accordance with the Declaration of Helsinki.

### 2.2. Image Analysis 

The angiograms were evaluated by a neuroradiologist with >15 years of experience in interventional radiology and 5 years of experience in mechanical thrombectomy, who was blinded to the patients’ clinical and treatment data. The images were rated according to the original definition of mTICI [7]. Alberta stroke programme early CT scores (ASPECTS) were calculated using automated software (e-ASPECTS–Brainomix, Suffolk, England). Embolization to a new territory (ENT) was defined as any occlusion related to the MT procedure outside the primary affected vessel territory. Hemorrhagic complications were assessed on follow-up computed tomography images obtained from each patient 24 ± 1 h after EVT, and were graded using the European Cooperative Acute Stroke Study III (ECASS III) criteria [8].

### 2.3. Thrombectomy Technique

In SR-only cases, the procedure consisted of introducing a large (minimum 0.70′′) internal diameter guiding catheter (Envoy 6F DA XB or Chaperon 6F) into extracranial ICA as high as possible, and then navigating with a microcatheter and a J-shaped microguidewire to pass the site of occlusion, placing a selected stent retriever (pReset–Phenox GmnH, Bochum, Germany or Catch–BALT Montmorency, France or Solitaire- Medtronic, Dublin, Ireland) and retrieving the thrombus with the aid of the 50cc vacuum-locked syringe attached to the guiding catheter.

In cases when the combined approach was used, the procedure consisted of introducing a long 8F (Neuron Max, Penumbra Inc., Alameda, CA, USA) sheath through an 8F short introducer in the common femoral artery. The long-sheath was put into extracranial ICA as high as possible, then a system consisting of an aspiration catheter (ACE-Penumbra Inc., Alameda, CA, USA), a microcatheter, and J-shaped microguidewire was introduced, with the microcatheter passing the occlusion site. Together with placing a selected stent retriever (pReset–Phenox GmbH, Bochum, Germany or Catch–BALT Montmorency, France or Solitaire-Medtronic, Dublin, Ireland or p3d-Penumbra inc., Alameda, CA, USA), the aspiration catheter (with continuous aspiration) was pushed to the proximal end of the clot, then the microcatheter was removed to increase the working lumen of the system. In the final step, the clot was pinched by pulling a part of the SR into the AC, and the whole system (SR + AC) was removed with the aid of aspiration of the 50cc syringe or a mechanical pump from the AC, along with the long sheath. In ideal conditions, this procedure is similar to the SAVE technique [5,6]. In some cases, this technique was modified, as dictated by requirements of the case. For example, in the case of very difficult and time-consuming navigation to the site of occlusion, usually only SR was removed, thus leaving the distal end of the AC in place for any further passes. In cases of M2 occlusions, unless the branch was large enough to accommodate AC, the AC was usually pushed to the distal part of the M1 segment, the aspiration was started, and the SR with the clot was pulled into M1, pinched in the AC, and then removed together.

All the procedures were performed by neuroradiologists with no less than 5 years’ experience in mechanical thrombectomy, and each of them performs > 70 MTs per annum.

### 2.4. Statistical Analysis

The aim of this observational, single-center retrospective study is a comparison of the simple stent retriever-based mechanical thrombectomy with the combined approach using stent retrievers and aspiration catheters. The assignment to treatment was at the discretion of the performing physician, but most of the patients treated with SO were recruited until the end of 2019, whilst cases from 2020 were treated mostly with CA. The primary hypothesis was that CA is superior to the SO technique. To test this hypothesis, we assessed the primary endpoint-frequency of complete reperfusion, defined as mTICI grade 3 after the first pass of the device. The secondary endpoints were as follows: the procedure success, defined as mTICI grades 2b-3; duration of the procedure, defined as time from groin puncture to obtaining the final angiogram; the total number of device passes; and the clinical outcome. The clinical outcome was measured by Δ NIHSS, defined as the difference between National Institutes of Health Stroke Scale (NIHSS) score at patients’ admission and discharge (excluding fatal outcomes) and 90 days’ modified Rankin Scale (mRS) score. The 90-days mRS was evaluated by a neurologist participating in the study during a patient’s follow-up visit in the outpatient clinic or, if not possible, during a telephone conversation with the patient or his/her caregiver. The normality of distribution of the continuous data was assessed using the Shapiro–Wilk test. In the case of normal distribution, the independent t-test was used to compare both groups. If data were not normally distributed, the Mann–Whitney U test was used. Fischer’s exact test was used to compare categorical data. *p* ≤ 0.05 was considered statistically significant.

## 3. Results

### Study Population

A total of 70 patients were included in this study. Baseline characteristics of the study population are shown in Table 1. Out of the 70 patients included, 33 were treated with stent retriever only, and 37 patients with stent retriever and aspiration-catheter. The characteristics of the study population are presented in Table 1.

There were no statistical differences in the population subgroups regarding the following: occlusion site, age, sex, ASPECTS and NIHSS at admission, comorbidities, ivTPA administration, and time from symptoms onset to thrombectomy.

**Figure 1:**
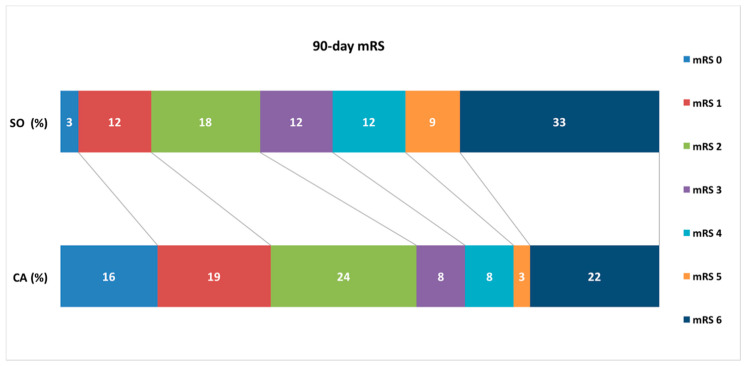
90-day modified Rankin Scale (mRS) in relation to a method of thrombectomy (% of patients in each group). SO = stent retriever only, CA = combined approach.

Results of the study endpoints’ analysis are presented in Table 2. There was a significant difference (*p* = 0.016) between the groups regarding first-pass success rate, with mTICI 3 of 46% in the CA group and 18% in the SO group, with OR of 3.83. The procedure success, described as final mTICI 2b or more, was also more frequent in the CA group than in the SO group—94.6% vs. 84.8% with OR of 3.13, yet the difference did not reach statistical significance (*p* = 0.193). The combined approach required a lower number of passes than the stent retriever only method (mean 1.76 vs. 2.09 passes per procedure), although it was not statistically significant (*p* = 0.114). Regarding mean duration of the procedure, the analysis showed that CA resulted in significant procedure time shortening—mean was 49 min in the CA group vs. 64 min in the SO group, *p* = 0.017. There was also a significant difference in clinical outcomes, with higher Δ NIHSS (9.3 in the CA group vs. 6.7 in the SO group, *p* = 0.025) after the procedure and 90-day mRS (median 2 in the CA group vs. 4 in the SO group, *p* = 0.031). No statistical significant differences were observed in the frequency of post-intervention SAH or symptomatic ICH and ENT. The 90-day mRS distribution in both groups of patients, with a significant shift towards better outomes in the CA group, is presented in Figure 1.

## 4. Discussion

In recent years, mechanical thrombectomy has become the standard of treatment in LVO [1,2,3,4,9,10]. There are two main methods of clot extraction from the affected vessel—either using a stent retriever or an aspiration catheter. It has been proven that both methods have comparable efficacy [4]. Each of the aforementioned techniques has its strengths and weaknesses. The devices used differ regarding their intra-arterial navigability and the way they engage with the clot [11,12]. Previous studies have shown that different techniques combining stent retrievers with aspiration catheters, such as SAVE, Solumbra or ARTS, present with high rates of reperfusion success and good clinical outcomes [5,6,13,14,15,16,17]. In 2019, Brehm et al., in a direct comparison, proved the superiority of SAVE over ADAPT in achieving faster and better recanalization with fewer device passes [18].

Complete and fast reperfusion is widely considered to be crucial for favorable outcomes [19,20,21]. CA in the presented study tends to appear better overall than SO-MT in achieving the procedure’s success, which was defined as final TICI > 2b (94.6% vs. 84.8%; *p* = 0.193). We put special stress on its first-pass success rate (CA 46% vs. SO 18%; *p* = 0.016), as previous papers demonstrated that there is a significant correlation between first-pass complete reperfusion and favorable outcomes [22,23]. It must be noted that our SO protocol did not require the use of balloon guiding catheters (BGC), and all the SO procedures were performed only with the aid of proximal aspiration from the large bore guiding catheters. This may have affected the efficacy of SO-MT, as several retrospective and observational studies have shown that the use of BGC during MT may be associated with better first-pass complete reperfusion rates, shorter procedure times, and better outcomes [24,25,26].

Combining AC with SR might help to overcome this limitation of our SO protocol; yet, switching to a more complex method of treatment raised certain concerns that it would be also more time consuming. It required a certain degree of adaptation from the operators, but, in most cases, the deployed stent retriever significantly facilitated navigation with the aspiration catheter. Finally, the CA resulted in a 15 minute reduction in the mean procedure duration (*p* = 0.017). We attribute this reduction mostly to the 2.5 times better first-pass efficacy of the CA in achieving TICI 3, which also resulted in fewer device passes needed to successfully complete the procedure.

Although TICI 2b is considered to be a relatively good angiographic result, we always perform subsequent passes in an attempt to achieve TICI 3. Repeat attempts may, however, increase the likelihood of device-related complications, such as arterial endothelial injury, ENT, parenchymal hematoma, and distal embolizations [23,27]. The OR for ENT, sICH and SAH in our study was higher in the SO group than in the CA group, yet these differences did not reach statistical significance.

Angiographic results in both groups corresponded well with the clinical outcomes. Patients treated with the CA had significantly better and immediate (during hospital stay) clinical improvement, measured with Δ NIHSS (9.3 in the CA group vs. 6.7 in the SO group, *p* = 0.025), and had better 90-day mRS scores (median 2 in the CA group vs. 4 in the SO group; *p* = 0.031). A total of 59% patients in the CA group achieved the state of functional independence (mRS score 0–2), whereas, in the SO group, this rate was only 33%. The shift in clinical outcomes is best visualized in Figure 1. These results are in accordance with those reported in other papers [5,6,13,18,19,22,23,28].

It is clear that the combined approach requires more angiographic equipment. Switching from SO to CA increased direct costs of mechanical thrombectomies in our institution by about 30%. This may raise concerns as to the cost effectiveness of the entire treatment. We did not perform such calculations, but a recently published paper, analyzing economic aspects of mechanical thrombectomy in the USA and EU, showed that patients with successful first-pass recanalization achieved better clinical outcomes, were discharged earlier from hospital, and required less care in the first year after stroke, all of which resulted in lower healthcare resource use and improved overall economic outcomes [28].

The major weakness of the presented study is its retrospective and observational design. There is also a possibility that better neurointerventional experience might have a positive impact on results in the CA group, although this impact may be mitigated by the learning curve during implementation of the new method.

Future preferably randomized controlled trials are necessary to the compare efficacy and safety of major methods of recanalization in stroke: aspiration catheters vs. stent retrievers vs. combined approach, with or without BGC.

## 5. Conclusions

Combining stent retrievers with aspiration catheters may offer beneficial effects for angiographic results and clinical outcomes in stroke patients undergoing endovascular treatment.

## Figures and Tables

**Table 1:** Baseline characteristics of the study population.

Parameter	Overall (*n* = 70)	SO (*n* = 33)	CA (*n* = 37)	*p* Value
Site of occlusion				0.262
Carotid	13	5	8	
M1	42	18	24	
M2	15	10	5	
Age (yrs ± SD)	71.6 *±* 13.4	74.2 *±* 10.2	69.2 *±* 15.6	0.061
Male sex	31	14	17	0.813
Atrial fibrillation	30	15	15	0.810
Diabetes	19	8	11	0.788
Arterial hypertension	57	27	30	1.000
Coronary heart disease	35	18	17	0.632
Dyslipidemia	31	15	16	1.000
ASPECTS: median, (IQR)	8, (7–9)	8, (7–9)	8, (7–8)	0.873
NIHSS on admission: median, (IQR)	17, (14–21)	17, (14–21)	16, (14–19)	0.191
Symptoms to thrombectomy (mean ± SD)	4:17 *±* 1:10	4:16 *±* 1:25	4:17 *±* 0:53	0.476
iv-rTPa	42 (60%)	20 (60.6%)	22 (59.5%)	1.000

SO = stent retriever only; CA = Combined approach; M1, M2 = M1, M2 segments of middle cerebral artery; SD = standard deviation; ASPECTS= Alberta stroke programme early CT scores, IQR = interquartile range; NIHSS = National Institutes of Health Stroke Scale; iv-Rtpa = intravenous recombined tissue plasminogen activator.

**Table 2:** Results of the study endpoints analysis.

Goal	SO	CA	OR (95% CI)	*p* Value
First pass mTICI 3	6 (18%)	17 (46%)	3.83 (1.28–11.44)	0.016
Procedure success— mTICI 2b-3	28 (84.8%)	35 (94.6%)	3.13 (0.56–17.34)	0.193
Total number of passes needed	2.09	1.76	*N*/A	0.114
Mean duration of the procedure (minutes ± SD)	64 ± 34	49 ± 21	*N*/A	0.017
90-day mRS: median, (IQR)	4, (2–6)	2, (1–4)	*N*/A	0.031
Δ NIHSS (± SD) (excluding deaths)	6.7 ± 4.0	9.3 *±* 5.1	*N*/A	0.025
Post interv SAH	2	1	0.43 (0.04–4.98)	0.499
sICH	3	5	1.56 (0.34–7.11)	0.564
ENT	4	2	0.41 (0.07–2.43)	0.328

SO = stent retriever only; CA = Combined approach; OR = odds ratio; CI = confidence interval; mTICI = modified Thrombolysis in Cerebral Infarction scale; SD = standard deviation; IQR = interquartile range; mRS = modified Rankin Scale; NIHSS = National Institutes of Health Stroke Scale; SAH = subarachnoid hemorrhage; sICH = symptomatic intracranial hemorrhage; ENT = embolization to a new territory.

## Data Availability

The data presented in this study are available on request from the corresponding author. The data are not publicly available due to privacy restrictions.
